# Anæmia

**Published:** 1906-02-03

**Authors:** 


### ANÆMIA.

**Anæmia and the Menstrual Function.**—Anæmia with its most obvious features of pallor, breathlessness, dyspepsia, and constipation, is excessively common among girls and young women, and is usually associated with more or less disturbance of menstruation. Whilst menorrhagia is sometimes present, a relative or absolute amenorrhœa is the rule, and the treatment of both conditions usually is the treatment of the anæmia itself. The subject has recently been considered by W. E. Fothergill,[1] who recalls the modern theories concerning the physiology of menstruation and ovulation, and points out that to the biological and physiological studies of Herbert Spencer is due the overthrow of the old popular "plethora" theory. In a living body chemical changes of two types are in constant evolution: constructive or anabolic changes whereby, from the comparatively simple ingesta, the complex living tissues are built up, and destructive or katabolic changes by which, as the result of living activity, simple chemical substances such as urea and carbon dioxide are produced. Where these metabolic processes are equal, and the breaking down and building up of tissue balance one another, body weight is stationary, and in the adult male of stationary weight these changes are balanced. But in the female there is a tendency to the production of an anabolic surplus available for the perpetuation of the race, only apparent in the lower mammals during pregnancy and lactation, but evidenced in the human species by the periodic menstrual discharge. As regards the mechanism of the function the recent experiments of Fraenkel and others show that it depends on the formation of a healthy corpus luteum after ovulation has occurred. The corpus luteum is, in fact, now regarded as a gland which produces the internal secretion of the ovary, without which secretion menstruation cannot occur nor pregnancy continue. In this view, then, the chemical stimulus of the secretion of the corpus luteum acts on the nervous centre for menstruation, and, if any anabolic surplus is present, leads to its discharge. The two factors necessary for menstruation are thus an anabolic surplus of blood and a healthy corpus luteum. Anæmia from any cause on the one hand, or interference with the normal development of a Graafian follicle on the other, as, for example, from ovarian new growth, may lead to amenorrhœa. Menorrhagia as a symptom of anæmia must be differently explained. It is probably due to inadequacy of the uterine muscle which, by compressing the uterine blood-vessels, controls the vascular supply of the organ and also the escape of blood from its lining membrane. Where the growth of muscle is not equal to the development of the blood-vessels hæmorrhage may be profuse, as occasionally happens at puberty. The menorrhagia of the menopause may also be explained in some instances by the physiological atrophy of the uterine muscle being in advance of that of the vessels. A similar explanation will account for the menorrhagia apt to follow an exhausting labour, and for that sometimes observed in acute specific fevers, the toxins of which may affect the muscle of the uterus as they do that of the heart and other viscera. Turning again to the function of the corpus luteum, Fothergill says that it has frequently been shown that if, during abdominal operations on women, the corpus luteum is excised or cauterised the next menstruation does not appear, but the function is re-established later after the formation of another corpus luteum. It has also been shown that if these bodies are excised in pregnant rabbits gestation comes to an end. It seems possible, therefore, that in some instances abortions may be due to interference with the full development of these organs, and that a failure to develop may be the actual cause of some cases of *sterility*, a subject recently dealt with in a lecture by A. R. Simpson,[2] who, before reviewing all the possible local causes, acknowledges that in some cases no local mischief or abnormality can be detected, and that the reason may lie in some constitutional defect such as malnutrition, obesity, or nerve-exhaustion. The charge that the over-intellectual development of American women accounts for their childlessness has been rebutted by the observations of Mary D. Jones, who points out that over-exertion in brain work is not more liable than over-exertion in any other line, as in muscular occupations, to lead to this result.

[1] Med. Chron., July. [2] Prac., July.

